# Decreased Level of Neurotrophic Factor Neuritin 1 in Women with Ovarian Endometriosis after Receiving Gonadotropin-Releasing Hormone Agonist Treatment

**DOI:** 10.3390/ijms20184352

**Published:** 2019-09-05

**Authors:** Endah Rahmawati, Wei-Chung Vivian Yang, Yen-Ping Lei, Pawan Kumar Maurya, Huei-Wen Chen, Chii-Ruey Tzeng

**Affiliations:** 1 Graduate Institute of Clinical Medicine, College of Medicine, Taipei Medical University, Taipei 11031, Taiwan; 2 Department of Obstetrics and Gynecology, Faculty of Medicine Public Health and Nursing, Universitas Gadjah Mada, Yogyakarta 55281, Indonesia; 3 The PhD Program for Translational Medicine, College of Medical Science and Technology, Taipei Medical University, Taipei 11031, Taiwan; 4 Department of Obstetrics and Gynecology, College of Medicine, Taipei Medical University, Taipei 11031, Taiwan; 5 Department of Biochemistry, Central University of Haryana, Mahendergarh 123031, India; 6 Graduate Institute of Toxicology, College of Medicine, National Taiwan University, Taipei 10051, Taiwan; 7 Center for Reproductive Medicine, Department of Obstetrics and Gynecology, Taipei Medical University Hospital, Taipei 11031, Taiwan

**Keywords:** ovarian endometriosis, gonadotropin-releasing hormone agonist, GnRHa, neuritin 1, NRN1

## Abstract

This study aimed to investigate the effect of gonadotropin-releasing hormone agonist (GnRHa) treatment on the expression of neuritin 1 (NRN1) in women with ovarian endometriosis. We collected tissues and serum from women with endometriosis treated with (*n* = 45) or without (*n* = 37) GnRHa. NRN1 mRNA and protein levels were measured using qPCR and Western blot. Immunolocalization of NRN1 in endometriotic tissues was examined using immunohistochemistry. In addition, a follow-up study was carried out to monitor the serum level of NRN1 in patients before and after GnRHa treatment. Both mRNA (*p* = 0.046) and protein (*p* = 0.0155) levels of NRN1 were significantly lower in endometriotic tissues from patients receiving GnRHa treatment compared to the untreated group. Both epithelial and stromal cells of endometriotic tissues from untreated women with endometriosis exhibited stronger staining of NRN1 but not in those who were treated with GnRHa. The follow-up study showed that the serum level of the NRN1 concentration decreased significantly from 1149 ± 192.3 to 379.2 ± 80.16 pg/mL after GnRHa treatment (*p* = 0.0098). The expression of NRN1 was significantly lower in women with ovarian endometriosis treated with GnRHa. These results suggest that NRN1 may be a biomarker response to the effect of GnRHa treatment for patients with ovarian endometriosis.

## 1. Introduction

Endometriosis is defined as the presence of endometrial tissues and glands outside the uterine cavity [1]. Although its genomic landscape has been recently revealed [2], the pathobiology of endometriosis, especially the molecular changes that occur in response to hormonal therapy, remains poorly understood. Most women with endometriosis suffer from ongoing pain and infertility [3]. These symptoms may be diminished by hormonal therapies that suppress estrogen production [4]. A gonadotropin-releasing hormone agonist (GnRHa) can be effective in relieving pain and improving the fertility potential of women with endometriosis [4].

Although ovarian endometriosis leading to pain has been widely discussed, its mechanism remains unclear. Stimulation of neurite infiltration by endometriotic lesions suggests that specific growth factors or other molecules produced by the ectopic tissue are capable of interacting with nerve fibers [5,6,7]. Some studies have attempted to elucidate the role of nerve fibers in pain in women with endometriosis, but their direct relationship remains controversial. The presence of nerve fibers in the functional layer of the endometrium has been suggested to be a reliable method for diagnosing endometriosis [8,9,10,11], but other reports have suggested that there is no significant difference in the density of nerve fibers in the endometrial functional layer in women with and without endometriosis [12]. Some evidence has also revealed that nerve growth factor (NGF) and PGP9.5-immunoactive nerve fibers are expressed in ovarian endometriotic lesions and may be involved in the generation of pain in women with ovarian endometriosis [13,14,15,16].

Neuritin 1 (NRN1) is a neurotrophic factor that is also known as the candidate plasticity gene 15 [17]. NRN1 is expressed in various human tissues including the brain, placenta, lung, liver, heart, skeletal muscle and also in various human cancers [18]. NRN1 has a plethora of biological functions in the nervous system and in the pathogenesis of some diseases. In the nervous system, NRN1 has at least three known functions. First, it exerts a neurotrophic effect by regulating neurite outgrowth and elongating the opposing dendritic and axonal arbors [17,19]. Second, it stimulates the development of motor neuron axon arbors by promoting neuromuscular synaptogenesis [20]. Third, it has an anti-apoptotic effect by inducing BCL2 expression [21]. Beyond the nervous system, NRN1 is associated with the severity of emphysema and with liver maturation and regeneration [22,23].

A complementary DNA (cDNA) microarray study showed that the neuritin 1 (*NRN1*) gene is upregulated in the ectopic endometrium of women with endometriosis (Supplementary Table S1) [24], which suggests that NRN1 may be associated with this disease. However, the effect of hormonal treatment on NRN1 expression in women with endometriosis needs to be elucidated. This study was conducted to investigate the NRN1 expressions in tissue and serum of women with endometriosis following GnRHa treatment.

## 2. Results

### 2.1. Characteristics of the Study Population

The baseline characteristics of the patients in this study are shown in Table 1. The total number of women with endometriosis was 37 in the untreated group and 45 in the GnRHa-treated group. The age, BMI, dysmenorrhea, use of analgesics, history of smoking and alcohol consumption did not differ significantly between the groups (*p* > 0.05). Because GnRHa can induce amenorrhea, we assigned the phase of the menstrual cycle only in the GnRHa-untreated group. An additional 10 patients were recruited into the follow-up study of the NRN1 serum level changes that occurred after the treatment (Table 2).

### 2.2. NRN1 mRNA Expression in Endometriotic Tissues of Patients Treated with GnRHa

**Figure 1:**
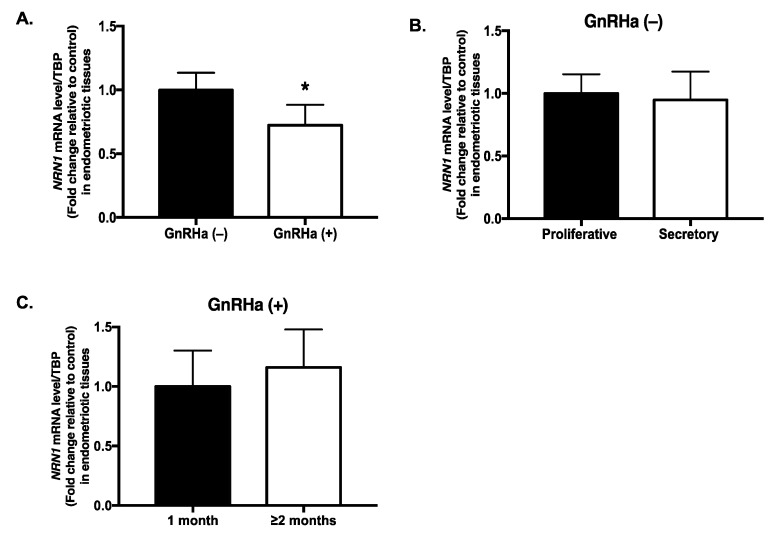
mRNA expression of Neuritin 1 (NRN1) in endometriotic tissues. (**A**) Expression of the NRN1 mRNA in patients treated with a gonadotrophin-releasing hormone agonist (GnRHa (+), *n* = 12) and in those not treated with a GnRHa (GnRHa (−), *n* = 14); * *p* < 0.05. (**B**) NRN1 mRNA levels in the GnRHa (−) group based on the menstrual cycle: the proliferative (*n* = 7) and secretory (*n* = 7) phases. (**C**) NRN1 mRNA expression in the GnRHa (+) group in patients who received treatment for 1 month (*n* = 3) and in those who received treatment for ≥2 months (*n* = 9). The TATA-box-binding protein gene was used as an internal control for qPCR. The data are expressed as the mean ± standard error of the mean. *n*, number of patients. The non-parametric Mann–Whitney U test was used for statistical analysis. Significance was set at *p* < 0.05.

The expression of the NRN1 mRNA was significantly lower (28%) in endometriotic tissues from the patients treated with GnRHa compared with the untreated group (*p* = 0.046) (Figure 1A). In addition, in the untreated group, NRN1 mRNA expression did not differ significantly between the proliferative and secretory phases of the cycle (*p* = 0.9) (Figure 1B). Moreover, NRN1 mRNA expression did not differ between patients who were treated with GnRHa for 1 month and those who were treated for ≥2 months (*p* = 0.86) (Figure 1C).

### 2.3. NRN1 Protein Expression in Patients Treated with GnRHa

**Figure 2:**
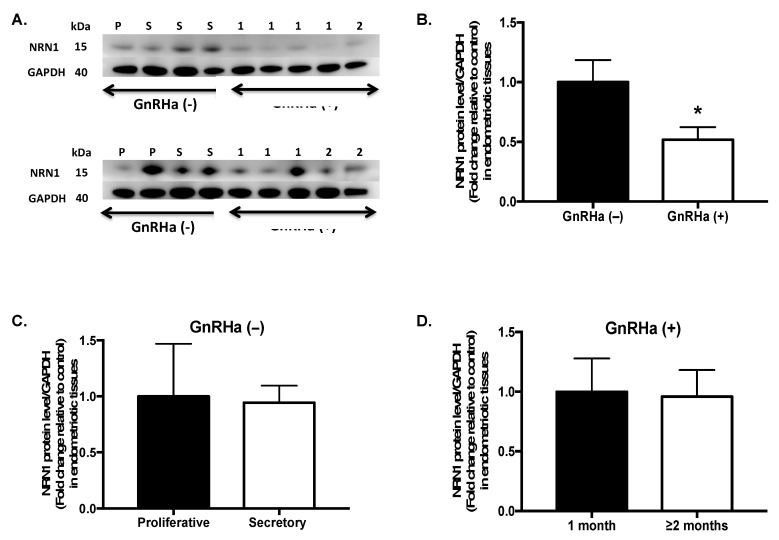
Expression of the NRN1 protein in endometriotic tissues. (**A**) Western blot images of NRN1 protein expression in patients treated with a gonadotrophin-releasing hormone agonist (GnRHa (+), *n* = 10) and in those not treated with a GnRHa (GnRHa (−), *n* = 8). P, proliferative; S, secretory; 1, patients treated for 1 month; 2, patients treated for 2 months. Glyceraldehyde 3-phosphate dehydrogenase (GAPDH) was the loading control. (**B**) The intensity of the NRN1 and GAPDH bands was quantified and is shown as a bar plot. * *p* = 0.0155. (**C**) NRN1 protein level in the GnRHa (−) group in the proliferative (*n* = 3) and secretory (*n* = 5) phases of the menstrual cycle. (**D**) The NRN1 protein level in the GnRHa (+) group in patients who were treated for 1 month (*n* = 7) and in those who were treated for ≥2 months (*n* = 3). The data are expressed as the mean ± standard error of the mean. *n*, number of patients. The non-parametric Mann–Whitney U test was used for statistical analysis. Significance was set at *p* < 0.05.

Expression of the NRN1 protein was consistent with the observation at the mRNA level. A Western blot analysis showed that the level of the NRN1 protein in endometriotic tissues was significantly lower (48%) in the GnRHa-treated group (*n* = 10) than in the untreated group (*n* = 8) (*p* = 0.015) (Figure 2A). The level of the NRN1 protein in the untreated group did not differ significantly between the proliferative and secretory phases of the cycle (*p* = 0.786) (Figure 2C). Moreover, expression of the NRN1 protein did not differ significantly between patients who were treated with GnRHa for 1 month and those who were treated for ≥2 months (*p* = 0.833) (Figure 2D).

### 2.4. Localization of NRN1 in Endometrial Tissues of Women with Endometriosis

**Figure 3:**
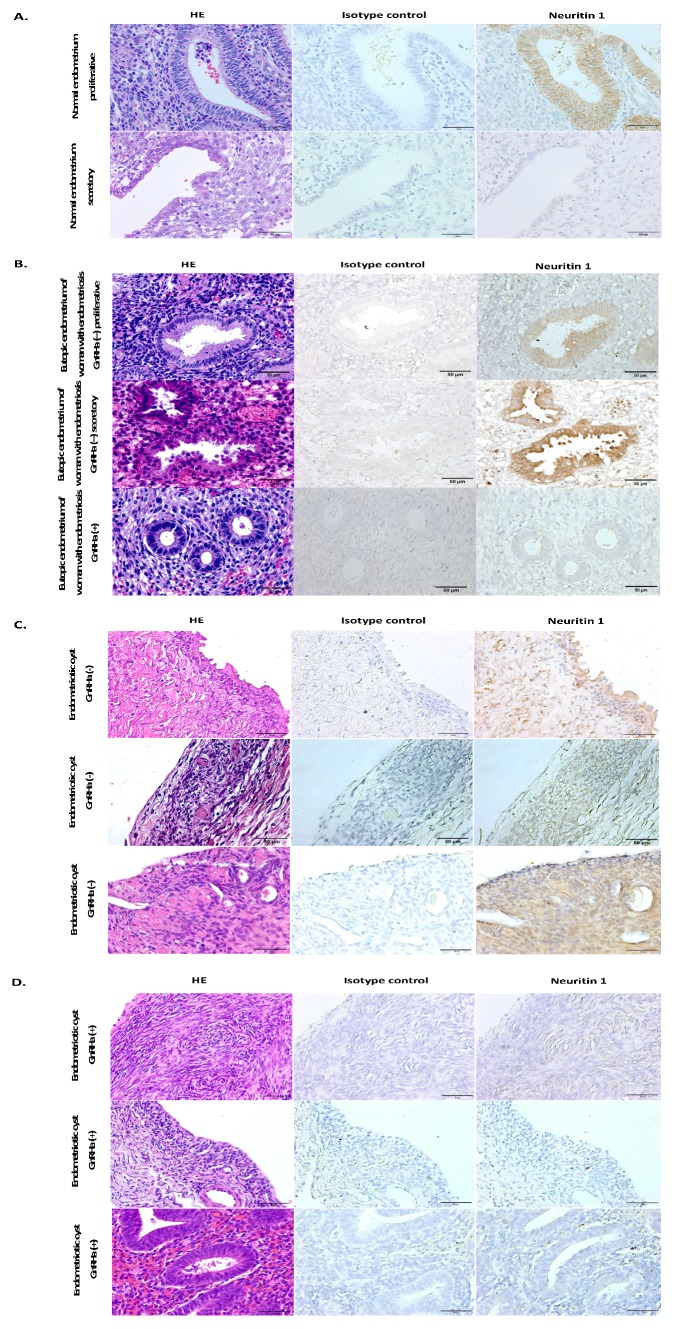
NRN1 protein expression and localization in endometrial tissues. Immunohistochemistry was used to localize the NRN1 protein in the normal endometrium (**A**), in the eutopic endometrium of women with endometriosis (**B**), and in endometriotic cysts with (**D**) and without (**C**) GnRHa treatment. Positive NRN1 staining is seen as a dark-brown precipitate on the surfaces of the cells. Hematoxylin and eosin staining were performed in each specimen to provide a tissue overview. Magnification, 400×; scale bar, 50 μm.

Immunohistochemistry (IHC) was used to evaluate the cellular localization and protein expression of NRN1 in endometrial tissues (Figure 3). Samples of the eutopic endometrium of women without endometriosis served as the control (Figure 3A). In the normal endometrium, NRN1 staining was detected only in the epithelial cells of the endometrial gland during the proliferative phase (Figure 3A), but nearly negative during the secretory phase (Figure 3A); negative staining was found in stroma cells both at proliferative and secretory phases (Figure 3A). In contrast, increased expression of NRN1 was found in the epithelial cells of the endometrial glands and stroma cells during both the proliferative and secretory phases in eutopic endometrium of the untreated women with endometriosis (Figure 3B). In the untreated endometriotic tissues, NRN1 expression was detected in both epithelial and stromal cells (Figure 3C). Decreased expression levels of NRN1 was observed in both endometrial glands’ epithelial and stromal cells in eutopic endometrium (Figure 3B) and endometriotic tissues (Figure 3D) of women with endometriosis who received GnRHa treatment.

### 2.5. Follow-Up Study

**Figure 4:**
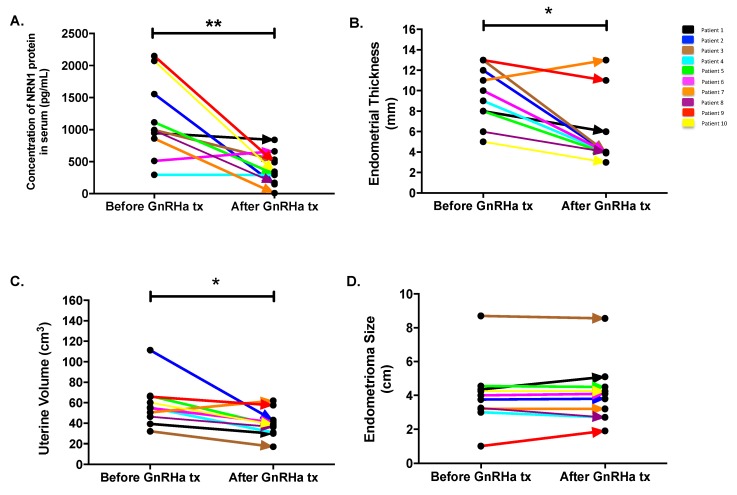
Serum level of NRN1, endometrial thickness, uterine volume and ovarian endometrioma diameter in women with endometriosis following GnRHa treatment. (**A**) NRN1 concentration was measured in the serum from 10 patients with endometriosis before and after GnRHa treatment by ELISA. The concentration of NRN1 in the serum decreased in nine out of the 10 patients. ** *p* < 0.01. (**B**) Endometrial thickness in women with endometriosis following GnRHa treatment; *n* = 10; * *p* *<* 0.05. (**C**) Uterine volume in women with endometriosis following GnRHa treatment, *n* = 10; * *p* < 0.05. (**D**) Ovarian endometrioma diameter in women with endometriosis following GnRHa treatment, *n* = 10; *p* = 0.875. The Wilcoxon signed-rank test was used for the analysis in this follow-up study. *n*, number of patients; tx, treatment. Significance was set at *p* < 0.05.

Follow-up monitoring of the NRN1 concentration in the serum of 10 patients showed a 67% decrease in the average concentration, from 1149 ± 192.3 to 379.2 ± 80.16 pg/mL after the GnRHa treatment (*p* = 0.0098). The serum concentration of the protein decreased in 9 out of the 10 patients (Figure 4A). Endometrial thickness was reduced by 40.1% (9.5 ± 0.885 to 5.69 ± 1.088 mm, *p* = 0.012) after GnRHa treatment (Figure 4B). Similarly, uterine volume shrank by 32.11% (58.32 ± 6.834 to 39.59 ± 4.114 mm; *p* = 0.014) following the GnRHa treatment (Figure 4C). Significant reduction of the ovarian endometrioma diameter was not found in patients after the GnRHa treatment for 1–2 months (4.005 ± 0.613 to 4.08 ± 0.584 cm; *p* = 0.875).

### 2.6. Serum Level of NRN1 in Women with Endometriosis

**Figure 5:**
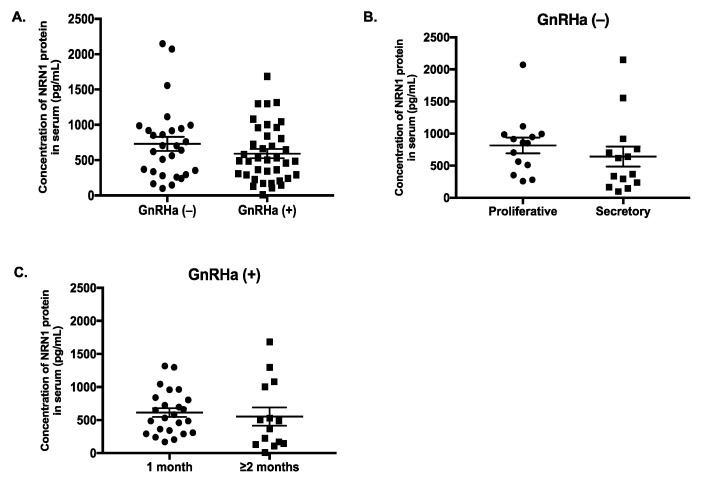
Level of NRN1 in serum of women with endometriosis was measured by ELISA. (**A**) Serum level of NRN1 in patients who were treated with gonadotrophin-releasing hormone agonist (GnRHa (+), *n* = 38) and in those who were not treated with the GnRHa (GnRHa (−), *n* = 28). (**B**) The level of NRN1 in the serum from the GnRHa (−) group in the proliferative (*n* = 14) and secretory (*n* = 14) phases of the menstrual cycle. (**C**) The level of NRN1 in the serum in the GnRHa (+) group in patients who were treated for 1 month (*n* = 24) and those who were treated for ≥2 months (*n* = 14). The non-parametric Mann–Whitney U test was used for statistical analysis. The data are expressed as the mean ± the standard error. Significance was set at *p* < 0.05. *n*, number of patients; tx, treatment.

The concentration of NRN1 in serum decreased by 19% on average from 729.3 ± 98.71 pg/mL in the untreated group to 591.4 ± 65.3 pg/mL in the GnRHa-treated group; however, this difference was not significant (*p* = 0.34) (Figure 5A). In addition, the level of NRN1 in the serum of the untreated patients did not differ between the proliferative (815.7 ± 122.6 pg/mL) and secretory (642.9 ± 155.8 pg/mL) phases (*p* = 0.12) of the menstrual cycle (Figure 5B). Moreover, the serum level of NRN1 did not differ significantly between patients who received GnRHa treatment for 1 month (613.7 ± 67.37 pg/mL) and for those who were treated for ≥2 months (553.2 ± 137.8 pg/mL) (*p* = 0.29) (Figure 5C).

## 3. Discussion

In this study, we investigated the expression of NRN1 in tissues and serum of women with endometriosis. Both mRNA and protein levels of NRN1 were downregulated in endometriotic tissues of the patients treated with GnRHa. The expression of several molecules with neurotrophic effects, including the nerve growth factor, neurotrophin-3 and the brain-derived neurotrophic factor, has been reported in women with endometriosis [25,26,27]. Our study showed that GnRHa treatment could also reduce the expression of neurotrophic factor, NRN1 in patients with ovarian endometriosis. Although the mechanism responsible for endometriosis-associated pain remains unclear, GnRHa has been proven to be effective in alleviating pain in women with endometriosis [28]. GnRHa treatment in women with perineural endometriosis restores the anatomical structure of the nerves and reduces pain symptoms [29]. Further study is needed to correlate the expression level of NRN1 and pain symptoms in women with endometriosis before and after GnRHa treatment.

We found significant decreases in the tissue levels of the NRN1, mRNA and protein in GnRHa-treated patients compared with the control group, but we did not find an equally significant decrease in the concentration of NRN1 in the serum of GnRHa-treated patients compared with the control group (Figure 5). This indicates that NRN1 expression may be determined by local metabolism in the tissue, rather than by its circulating serum levels. However, in the follow-up study, the concentration of NRN1 in the serum decreased significantly in the GnRHa-treated group (Figure 4A). Compared with measuring the serum level of NRN1 in the two different groups of patients (GnRHa-treated and -untreated groups), the monitoring of the serum levels of NRN1 before and after GnRHa treatment in the same patients will more likely reflect response to therapy.

Downregulation of the pituitary, suppression of gonadotropin release and achievement of the sustained hypo-estrogenic state are the principals of GnRHa treatment in women with endometriosis [30]. Our recent report showed that GnRHa induced lower estradiol level in women with endometriosis [31]. In this study, we found that GnRHa induced significant reduction in endometrial thickness and uterine volume of patients with ovarian endometrioma (Figure 4) as a response to the hormonal change. We did find the significant effect of GnRHa treatment on the shrinkage of ovarian endometrioma. The absence of a reduction in the size of endometrioma may be caused by GnRHa treatment that was only given for 1–2 months prior to laparoscopic surgery in patients with ovarian endometriosis. Several randomized control trials demonstrated the regression of endometriosis after six months of GnRHa treatment [30]. The pre-treatment using GnRHa prior to in vitro fertilization or intracytoplasmic sperm injection (IUI) in women with endometriosis for 3–6 months induces higher pregnancy rate [32,33]. Another study demonstrated that GnRHa treatment for three months improves the endometrial receptivity in women with endometriosis [34].

Considering the cyclical nature of the endometrium and the fact that endometriosis is an estrogen-dependent disease, we also studied whether the menstrual cycle affects the expression of NRN1. The IHC data obtained from normal endometrium showed that NRN1 was highly expressed in the endometrial gland epithelial cells during the proliferative phase of the menstrual cycle and was slightly expressed during its secretory phase (Figure 3A). A high level of estradiol during the proliferative phase may affect the expression of NRN1 in normal endometrium. In eutopic endometrium from untreated women with endometriosis, we found an unchanged level of expression of NRN1 both in the proliferative and secretory phases. Likewise, the menstrual cycle did not affect the NRN1, mRNA and protein levels (in endometriotic tissues and serum) of women with endometriosis. In contrast to the normal endometrium, estradiol appeared to be the dominating sex steroid in the two phases of the cycle, and there were no cyclical changes in estradiol levels in endometriotic tissues [35]. This fact may explain why there was no difference in NRN1 expression between the proliferative and secretory phases in patients with endometriosis. There is evidence that a gonadal steroid induced the expression of NRN1 in the motor neuron [36], whereas the question of how estradiol may induce NRN1 expression in endometriosis needs further investigation.

In this study, we only included ovarian endometriosis, while peritoneal and deep infiltrating endometriotic tissues excluded. The current study had a limited number of samples, therefore further studies with a larger sample size are required. In addition, studies observing the altered expression of NRN1 in the primary culture cells from endometriotic tissue before and after estradiol treatment are important to investigate whether GnRHa treatment directly affects the NRN1 expression.

This report demonstrated reduced expression of NRN1 in endometriotic tissues treated with GnRHa compared to untreated group and downregulation of NRN1 in the serum of women with endometriosis after GnRHa treatment. It suggests that NRN1 may be a potential biomarker reflecting the therapeutic efficacy of GnRHa treatment for endometriosis. The detailed regulatory mechanisms underlying the effect of GnRHa on NRN1 expression and the correlation between the expression of NRN1 and the pathogenesis of endometriosis require further studies.

## 4. Materials and Methods

### 4.1. Patient Recruitment and Specimen Collection

Patients who were scheduled for laparoscopic surgery for ovarian endometriosis were recruited to participate in this study from March 2011 to October 2016. None of the patients had previous laparoscopic surgery for endometriosis before enrolment in this study. The baseline characteristics of the patients are shown in Table 1. This study was approved by the Joint International Review Board of the Taipei Medical University Hospital, Taipei, Taiwan (TMU-JIRB 201006002 and TMU-JIRB 201305035), and each participant signed an informed consent form before the initiation of the study.

Samples were collected at two different times in two different groups of patients. In the first cohort, we initiated a pilot study by collecting ectopic endometrial tissues from women who had been treated (*n* = 12) and those who had not been treated (*n* = 14) with GnRHa to measure the NRN1 mRNA level. To confirm the results at the protein level, we recruited an additional 56 women with endometriosis who were treated with or without GnRHa, and collected endometriotic tissue and serum samples from these women. Only 18 out of the 56 women in the second cohort provided both tissue and serum samples; 10 provided only a tissue sample, and 28 provided only a serum sample. We also obtained normal endometrium from women without endometriosis and eutopic endometrium from women with endometriosis treated and untreated with GnRHa for immunohistochemistry (IHC) study.

In a follow-up study, serum samples were collected from 10 patients to compare the serum NRN1 concentration before and after GnRHa treatment (Table 2). Inclusion criteria for the follow-up study were the patients’ willingness to sign an informed consent twice: First, at recruitment in the outpatient clinic when pre-treatment serum sample was collected and second, before laparoscopic surgery when a post-treatment serum sample was collected. Ultrasonography was used to measure endometrial thickness, uterine volume and ovarian endometrioma diameter of each patient before and after GnRHa treatment in the follow-up study. The measurement of endometrium was done in the thickest part of the fundus on longitudinal images. Uterine volume was calculated using a formula that has been reported previously [37]. Ovarian endometrioma was assessed in two dimensions and the mean diameter was calculated. The duration of the GnRHa treatment (from GnRHa injection to the operating day) in the follow-up study ranged from 1 to 2 months. We also analyzed the expression of NRN1 in tissue and serum samples according to the duration of GnRHa treatment in the treatment group and NRN1 expression throughout the menstrual cycle in the untreated group.

The patients included in this study were confirmed to have ovarian endometriosis by visual laparoscopy and histology. All of them had moderate to severe endometriosis according to the classification of the revised guidelines of the American Society for Reproductive Medicine [38]. The phase of the menstrual cycle was assigned according to the last menstrual period. None of the patients in the untreated group had a history of any hormonal treatment, including contraceptive pills. In the treated group, we excluded women with endometriosis who received hormonal treatment other than a GnRHa (e.g., danazol, follicle-stimulating hormone or other hormonal drugs). The treatment dose of the GnRHa (leuprolide acetate; Lupron Depot, Takeda Pharmaceutical Company Ltd., Osaka, Japan) was 1.875 mg/injection/month subcutaneously injection.

All tissue specimens were collected at the time of laparoscopic resection, snap-frozen in liquid nitrogen, and kept at −80 °C until quantitative polymerase chain reaction (qPCR) and Western blot analysis was performed. For IHC, the specimens were immediately fixed in 10% paraformaldehyde for 12 h and embedded in paraffin according to standard protocols. The blood samples were collected via peripheral venipuncture as part of the pre-operative work-up (before anesthesia). Serum was extracted by centrifugation at 3000 rpm for 10 min and stored at −80 °C until use.

### 4.2. RNA Isolation and qPCR Analysis

*NRN1* expression was evaluated by qPCR using the SYBR Green PCR Master Mix (Applied Biosystems Inc., Carlsbad, CA, USA). Total RNA was isolated from endometriotic tissues from the treated and untreated groups using the TRIzol method (Invitrogen, Carlsbad, CA, USA). Assessment of both RNA concentration and the purity in the extracted samples was done using Nanodrop, and A260/A280 ratio was more than 1.9 for each sample. cDNA synthesis was performed with a reverse transcription kit (Invitrogen). The TATA-box-binding protein *(TBP)* gene was used as an internal control. The oligonucleotide primers that were used for the amplification of *NRN1* (forward: 5′-GCGACAGCATGGCCAACTAC-3′; reverse: 5′-CCTTCCTGGCAATCCGTAAGG-3′) and *TBP* (forward: 5′-TGCACAGGAGCCAAGAGTGAA-3′, reverse: 5′-CACATCACAGCTCCCCACCA-3′) were designed using the Primer Express v2.0 Software (Applied Biosystems Inc., Foster City, CA, USA). The relative expression level of *NRN1* was compared to that of the internal control. Experiments were performed in duplicate.

### 4.3. Western Blot Analysis

Western blot analysis was used to quantify the expression of the NRN1 protein in the endometriotic tissues. Endometriotic tissues were homogenized in mammalian tissue lysis buffer (CelLytic MT, Sigma-Aldrich, St. Louis, MO, USA). Protein quantification was performed using a Bio-Rad DC protein assay kit (Bio-Rad Laboratories Ltd., Mississauga, ON, Canada). Equal amounts of total protein (30 μg) from each sample were resolved in pre-cast polyacrylamide gels, subjected to electrophoresis and transferred onto a polyvinylidene fluoride membrane (GE Healthcare, Amersham, UK; 0.45 μm) using a semi-dry electrophoretic transfer cell (Bio-Rad Laboratories). After blocking, the membrane was probed with the following antibodies: rabbit anti-NRN1 (dilution, 1:500; ab64186; Abcam, Cambridge, UK) and mouse anti-glyceraldehyde 3-phosphate dehydrogenase (GAPDH) (dilution, 1:5000; ab8245; Abcam). GAPDH was used as the loading control. The membrane was then probed with the appropriate secondary antibody labeled with horseradish peroxidase. The bands were visualized using an enhanced chemiluminescence method and a BioSpectrum Imaging System (UVP, Upland, CA, USA). The density values of the bands were normalized to that of GAPDH.

### 4.4. Immunohistochemistry

IHC was used to localize the NRN1 protein qualitatively in eutopic endometrium and endometriotic tissues from women with endometriosis. IHC was performed using a Novolink Polymer Detection System (RE7140-K; Leica Biosystems, Newcastle Upon Tyne, UK). Paraffin blocks of each sample were cut at a thickness of 3 μm. Eutopic endometrial tissues from patients without endometriosis (normal endometrium) were used as control. Routine hematoxylin and eosin staining were performed to provide a tissue overview. Sections were deparaffinized and dehydrated, and antigen retrieval was performed in Novocastra Epitope Retrieval Solution, pH 6.0 (Leica Biosystems). The tissue sections were then processed using the kit according to the manufacturer’s protocol and were incubated with the primary antibody (rabbit polyclonal anti-NRN1 antibody; ab64186; dilution, 1:100; Abcam) overnight at 4 °C. To develop the peroxidase activity, sections were incubated with 3,3′-diaminobenzidine (DAB) working solution (Novocastra DAB Chromogen and Novolink DAB Substrate Buffer, Leica Biosystems) and counterstained with Novocastra Hematoxylin (Leica Biosystems). Negative controls were treated identically with the exception that the primary antibody was replaced with a rabbit IgG isotype control (ab199376; Abcam). Images were captured using the SPOT imaging software.

### 4.5. Enzyme-Linked Immunosorbent Assay

The concentration of the NRN1 protein was measured quantitatively using a commercially available enzyme-linked immunosorbent (ELISA) kit (MBS912929; MyBioSource, San Diego, CA, USA). The assay was performed according to the manufacturer’s instructions. Briefly, the samples were added to a 96-well plate that had been pre-coated with an anti-NRN1 antibody. The unbound proteins were removed by extensive washing and the bound NRN1 was detected using another biotin-conjugated anti-NRN1 antibody. After extensive washing, an avidin-conjugated horseradish peroxidase substrate was added into the wells for color development. The developed color was measured as an optical density value using a microplate reader at a wavelength of 450 nm, with the correction wavelength set at 540 nm. A standard curve for NRN1 was included in each plate and the NRN1 concentration of each serum sample was determined via interpolation using the standard curve. Each sample was analyzed in two independent experiments and the mean value was calculated.

### 4.6. Statistical Analysis

Statistical analysis was performed using GraphPad Prism version 7.00 for Mac (GraphPad Software, San Diego, CA, USA). Age and body mass index (BMI) were compared between the treated and untreated groups using an unpaired two-tailed *t*-test. Dysmenorrhea, analgesic use, smoking and alcohol consumption were compared using the chi-squared test. The non-parametric Mann–Whitney U test was used to compare the expression of NRN1 between the two groups. The Wilcoxon signed-rank test was used to compare the serum NRN1 concentration, endometrial thickness, uterine volume and ovarian endometrioma diameter before and after the GnRHa treatment. Significance was set at *p* < 0.05.

## Figures and Tables

**Table 1:** Baseline characteristics of the study population.

	Women with Endometriosis GnRHa (−)	Women with Endometriosis GnRHa (+)	*p*-Value
**Total (n)**	37	45	
**Age (years)**	32.4 ± 5.9 (21–44)	34.4 ± 4.2 (25–48)	0.08
**BMI (kg/m^2^)**	21.6 ± 3.8 (16–35)	20.9 ± 2.7 (16–29)	0.35
**Dysmenorrhea (%)**	30 (81.1%)	39 (86.7%)	0.49
**Analgesics (%)**	8 (21.6%)	14 (31.1%)	0.33
**Smoking (%)**	9 (24.3%)	17 (37.8%)	0.19
**Alcohol (%)**	5 (13.5%)	7 (15.6%)	0.79
**Proliferative phase**	16 (43.2%)		
**Secretory phase**	21 (56.8%)		

The data are expressed as the mean ± SD (range) for continuous variables and as the number of patients (%) for other variables. GnRHa, gonadotrophin-releasing hormone agonist; BMI, body mass index.

**Table 2:** Characteristics of women with endometriosis involved in the follow-up study.

Patients	Age (y)	BMI (kg/m^2^)	Duration of GnRHa Tx	Diagnosis at Laparoscopy	Endometrial Thickness (mm)		Uterine Volume (mm3)		Chocolate Cyst Size (cm)	
					Before GnRHa Tx	After GnRHa Tx	Before GnRHa Tx	After GnRHa Tx	Before GnRHa Tx	After GnRHa Tx
1	32	19.88	1	Stage IV endometriosis	8	6	39.421	30.055	4.35	5.1
2	33	22.04	2	Stage III endometriosis	12	4	111.368	43.156	3.75	3.8
3	32	20.57	2	Stage III endometriosis	13	4	32.236	17.199	8.7	8.55
4	38	18.61	1	Stage III endometriosis	9	4	55.561	31.063	3	2.7
5	40	20.83	1	Stage III endometriosis	8	3.9	66.695	38.322	4.55	4.5
6	33	20.78	1	Stage IV endometriosis	10	4	54.843	41.047	4	4.1
7	33	22.59	2	Stage III endometriosis	11	13	50.544	62.057	3.2	3.2
8	33	18.56	2	Stage III endometriosis	6	4	46.476	36.504	3.25	2.7
9	35	17.27	2	Stage IV endometriosis	13	11	65.869	57.678	1	1.9
10	32	23.05	1	Stage IV endometriosis	5	3	60.191	38.788	4.25	4.25

BMI, body mass index; GnRHa, gonadotropin-releasing hormone agonist; yr = year; tx = treatment.

## Supplementary Materials

Supplementary materials can be found at https://www.mdpi.com/1422-0067/20/18/4352/s1.

## Abbreviations

NRN1	Neuritin 1
GnRHa	Gonadotropin-releasing hormone agonist
cDNA	Complementary deoxyribonucleic acid
JIRB	Joint International Review Board
qPCR	Quantitative polymerase chain reaction
mRNA	Messenger ribonucleic acid
WB	Western blotting
IHC	Immunohistochemistry
ELISA	Enzyme-linked immunosorbent assay
TBP	TATA-box-binding protein
GAPDH	Glyceraldehyde 3-phosphate dehydrogenase
DAB	Diaminobenzidine
IgG	Immunoglobulin
