# Supplementary material for: Decreased Level of Neurotrophic Factor Neuritin 1 in Women with Ovarian Endometriosis after Receiving Gonadotropin-Releasing Hormone Agonist Treatment
Source: Int J Mol Sci. 2019 Sep 5;20(18):4352. doi: 10.3390/ijms20184352 (PMC6770869; doi:10.3390/ijms20184352)
Supplement: Supplementary file 1 [file ijms-20-04352-s001.pdf]

Supplementary Table 1. List of the genes were differentially expressed in the ectopic endometrium of patients with and without GnRHa treatment.

SYNPO	synaptopodin	LAMA4	laminin, alpha 4
MGP	matrix Gla protein	GIMAP6	GTPase, IMAP family member 6
EMCN	Endomucin	AEBP1	AE binding protein 1
FGL2	fibrinogen-like 2	ITGA7	integrin, alpha 7
SPARCL1	SPARC-like 1 (hevin)	VSNL1	visinin-like 1
RERGL	RERG/RAS-like	MEIS2	Meis homeobox 2
LDB3	LIM domain binding 3	FRY	furry homolog (Drosophila)
CLU	clusterin	SPEG	SPEG complex locus
EPHA3	EPH receptor A3	CPE	carboxypeptidase E
FRZB	frizzled-related protein	TNS1	tensin 1
CAV2	caveolin 2	PDLIM5	PDZ and LIM domain 5
CTSG	cathepsin G	PGM5	phosphoglucomutase 5
NRN1	neuritin 1	MEF2C	myocyte enhancer factor 2C
PLSCR4	phospholipid scramblase 4	TMEM47	transmembrane protein 47
Annexin 13	Annexin XIII	DS	Adhesion molecule (DS)
CDH 5	Cadherin 5	CDH 1	E-cadherin
CALD-1	Caldesmon 1	ITGA1	Integrin, alpha 1
CTNNAL-1	Catenin alpha-like1	MMP-14	Matrix metalloproteinase-14
CTSG	Cathepsin L2	CD 36	Cluster of Differentiation 36
TMP2	TIMP2	VCAM-1	Vascular cell adhesion molecule 1
COLEC12	collectin sub-family member 12		
RGS5	regulator of G-protein signaling 5		
AGTR1	angiotensin II receptor, type 1		
HLA-DPA1	major histocompatibility complex, class II, DP alpha 1		
MCTP1	multiple C2 domains, transmembrane 1		
PDE1A	phosphodiesterase 1A, calmodulin-dependent		
DARC	Duffy blood group, chemokine receptor		
ITM2A	integral membrane protein 2A		
PDGFA	platelet-derived growth factor alpha polypeptide		
PTGIS	prostaglandin I2 (prostacyclin) synthase		
SERPINI1	serpin peptidase inhibitor, clade I (neuroserpin), member 1		
FAM129A	family with sequence similarity 129, member A		
CCDC69	coiled-coil domain containing 69		
ATP2A3	ATPase, Ca++ transporting, ubiquitous		
SNCG	synuclein, gamma (breast cancer-specific protein 1)		
SORBS1	sorbin and SH3 domain containing 1		
ADH1B	alcohol dehydrogenase 1B (class I), beta polypeptide		
ITM2A	integral membrane protein 2A		
VGLL3	vestigial like 3 (Drosophila)		
FMO1	flavin containing monooxygenase 1		
CCL21	chemokine (C-C motif) ligand 21		
LHFP	lipoma HMGIC fusion partner		
FHL5	four and a half LIM domains 5		
HSD17B6	hydroxysteroid (17-beta) dehydrogenase 6 homolog (mouse)		
PDE4DIP	phosphodiesterase 4D interacting protein		
